# Embodiment in a Child-Like Talking Virtual Body Influences Object Size Perception, Self-Identification, and Subsequent Real Speaking

**DOI:** 10.1038/s41598-017-09497-3

**Published:** 2017-08-29

**Authors:** Ana Tajadura-Jiménez, Domna Banakou, Nadia Bianchi-Berthouze, Mel Slater

**Affiliations:** 1 UCL Interaction Centre (UCLIC), University College London, London, UK; 2 Universidad Loyola Andalucía, Department of Psychology, Seville, Spain; 3 Universidad Loyola Andalucía, Human Neuroscience Lab, Seville, Spain; 4 Event Lab, Department of Clinical Psychology and Psychobiology, Faculty of Psychology, Barcelona, Spain; 5 Institute of Neurosciences, University of Barcelona, Barcelona, Spain; 6 Institució Catalana de Recerca i Estudis Avançats (ICREA), Barcelona, Spain; 7 Department of Computer Science, University College London, London, UK

## Abstract

People’s mental representations of their own body are malleable and continuously updated through sensory cues. Altering one’s body-representation can lead to changes in object perception and implicit attitudes. Virtual reality has been used to embody adults in the body of a 4-year-old child or a scaled-down adult body. Child embodiment was found to cause an overestimation of object sizes, approximately double that during adult embodiment, and identification of the self with child-like attributes. Here we tested the contribution of auditory cues related to one’s own voice to these visually-driven effects. In a 2 × 2 factorial design, visual and auditory feedback on one’s own body were varied across conditions, which included embodiment in a child or scaled-down adult body, and real (undistorted) or child-like voice feedback. The results replicated, in an older population, previous findings regarding size estimations and implicit attitudes. Further, although auditory cues were not found to enhance these effects, we show that the strength of the embodiment illusion depends on the child-like voice feedback being congruent or incongruent with the age of the virtual body. Results also showed the positive emotional impact of the illusion of owning a child’s body, opening up possibilities for health applications.

## Introduction

How we mentally represent our body in terms of its physical appearance and its capabilities for action is vital in creating a sense of self-identity, and for acting and interacting with the world and with others^1–3^. Importantly, over the past 20 years, extensive research has shown the malleability of our brain’s body representations. It is now apparent that these body-representations update continuously through the incoming sensory information related to one’s own body^4–7^. One of the most famous examples demonstrating this sensory-driven malleability of body-representations is that of the Rubber Hand Illusion (RHI). In this paradigm, participants experience a rubber hand placed in front of them as part of their own body when they observe the rubber hand being touched while receiving synchronously and corresponding touch on their own, unseen, hand^8^. If the rubber hand is threatened, this evokes physiological and cortical responses in participants similar to those expected if their own real hand were threatened^9,10^. Analogous techniques have been used to generate illusions of ownership over another person’s face (the *enfacement illusion*^11–13^) and also over a whole mannequin body, seen from first person perspective (1PP) through a head-mounted display^14^. Such bodily illusions have been reproduced in virtual reality (VR), both with respect to a virtual arm^15,16^, or a full virtual body seen to substitute the real one^17,18^. A threat to an arm belonging to the embodied virtual body results in corresponding behavioral^16^ and cortical^19^ responses as if the real arm had been attacked. Moreover, when an embodied virtual arm is made invisible (as if amputated) then the evidence suggests that there is decreased corticospinal activity^20^.

Furthermore, a number of experimental studies suggest that altering one’s body - representations can lead to changes in cognition and perception of other people and objects in the environment. For example, inducing the RHI over a black rubber hand has been shown to lead to a reduction of implicit racial bias in Caucasian participants^21,22^. The same has been demonstrated with respect to a dark-skinned face being observed during the enfacement illusion^23^, or with respect to a full dark-skinned virtual body seen from a 1PP^24^. This effect has also been shown to last at least one week^25^.

In a study using VR, Banakou *et al*.^26^ embodied adults in a virtual body representing a 4-year-old child, and also in an adult body though shrunk down to the size of the child. It was found that inducing the illusion of ownership over a body of a child in adults influences the adult-child categorizations of self, compared to others. Further, it was found that both groups overestimated object sizes, but those in the child body by approximately double the extent of those in the adult body. Changes in the perception of object sizes resulting from changes in the size of the owned body are in line with previous findings showing that adults overestimate object sizes when embodied in a small mannequin (a ‘barbie doll’) and underestimate them when embodied in a giant body^27^. However, here it was shown that not only size accounted for this change in perception, but also the shape of the body (child or adult).

While these and the majority of studies on the plasticity of body-representation have manipulated visual, proprioceptive, tactile, and motor cues in order to elicit illusions of body ownership and agency over a virtual body, a few recent studies have shown that auditory cues can also lead to the induction of bodily illusions under specific conditions. In particular, these studies have investigated the effects of altering the sounds produced by one’s body when interacting with other objects in the environment. Results have shown, for example, that alterations in the sounds that one’s hand produces when tapping a surface may result in updates in the represented arm length^28,29^ and/or strength^30^. Further, real-time alterations of the frequency spectra of the sounds people produce while walking have been shown to change people’s perception of their own body weight, and lead to a related gait pattern and emotional state^31^. Previous studies have also revealed that sound may provide information about material properties of the body. For instance, changes in the frequency spectra of the sounds produced when rubbing one’s hands together can alter the perceived skin moisture and dryness, known as the “parchment-skin” illusion^32,33^. A related study showed that altering the sound produced when a hammer hits one’s hand, so that it sounds as a hammer hitting marble, makes one’s hand feel stiffer and heavier^34^.

Here we investigate the influence of a different type of sound on body-representation. This is related to peoples’ own vocalizations, which play an important role in representing one’s body, as these vary greatly across individuals and carry information about the age, gender, and body size of the speaker^35^. In particular, the aim of the present study was to quantify the extent to which real-time morphing of an adult’s own voice, so that it sounds as a child-like voice, enhances the illusion of ownership over a virtual child body experienced from a 1PP in VR (see related work by Deutschmann *et al*.^36^), in which voice-morphing was used to constitute a self-virtual body of a different gender as one’s own. We also addressed the question as to how this embodiment might influence size-perception of the environment and the categorizations of self, compared to others^26^.

When we hear the voice of an unknown speaker we can often rapidly tell whether it comes from an adult, a young speaker, or a child, or whether it comes from a woman or man even when we are not looking at the speaker directly (e.g., when the speaker is out of view or on the phone). These judgments are made based on the acoustic cues of the voice signal. Two of these highly salient cues are linked to the frequency of the voice signal, which in turn depends on differences in the mass of the vocal folds and the length of the vocal tract: voice pitch and formant frequencies^35^. Voice pitch changes according to the opening/closing rate of the vocal folds (glottal-pulse rate)^35^. The average 4-year old’s fundamental frequency is around 240–260 Hz, with small sex differences, while the average fundamental frequency for men is around 120 Hz and for women around 200 Hz^37–40^. Formant frequencies refer to the frequency of the prominent spectral peaks in the voice signal and they shift according to the vocal-tract length (VLT) of the speaker, with longer vocal-tracts resulting in higher shifts of the formant frequencies towards lower frequencies^41^. Young children have the shortest VLTs, while adult men have the longest VLTs, and adult women have intermediate VLTs^41,42^. On average, taking as reference the formant frequencies of children aged 4 years, the formant frequencies of adult males decrease around 32% and those of adult female decrease around 20%^35,38^. Previous studies have shown that, in normal speech, listeners’ judgments of an unknown speaker’s age are influenced about equally by voice pitch and formant frequencies^35^, and that listeners can reliably discriminate changes in voice pitch of about 2% and changes in formant frequencies of 6–10%^43^. While these prior studies explored the effects of passive listening to speech sounds in the perceived gender and age of an unknown speaker, our study focuses on changes in the perception of one’s own body due to altering self-produced speech sounds.

The experiment that we describe here was conducted on participants over 40 years old. In order to induce a level of subjective body ownership over a life-sized virtual body seen from 1PP we used the technique of visuomotor synchrony following earlier successful applications^26,44^. Participants wore a full body motion capture suit that tracked their movements in real-time. Through a wide field-of-view head-tracked stereo head mounted display they would see their virtual body directly when looking down towards their real body and also in a virtual mirror. We compare embodiment in a child with real undistorted or child-like voice feedback with embodiment in that of a scaled – down adult body of the same size, also with real undistorted or child-like voice feedback. We consider five issues: First, whether there is evidence of an illusion of body ownership with respect to these two virtual bodies, as suggested by earlier findings that were with younger adults, and in particular whether the strength of the illusion differs for each of the auditory feedback cues. Second, we considered whether adults might come to experience a child-like voice as their own voice as a result of experiencing the illusion of ownership over a child virtual body. This is related to the findings of Banakou and Slater^44^ who showed that embodiment of adults in a virtual body that speaks leads to illusory agency over the speaking. Third, whether perception of sizes of objects is influenced by the virtual body form and audio cues. Fourth, whether there is a difference in reaction times in attributing child-like or adult-like attributes to the self, following the body form and audio feedback. Last, we also investigated the potential impact of the illusion of owning a child’s body on feeling younger and on emotional state. Given the widespread preoccupations with aging-related changes and with maintaining younger looks, which are higher in women than in men^45,46^, we were interested to find whether there would be a positive impact of the illusion of having a child’s body on emotional state.

## Materials and Methods

### Ethics

The experiment was approved by Comissió Bioètica of Universitat de Barcelona. All participants gave their written informed consent prior to participating. The study was performed according to institutional ethics and national standards for the protection of human participants. Ethical considerations included informed consent, right to withdraw, and confidentiality. Exclusion criteria were epilepsy, use of medication, recent consumption of alcohol, significant back or neck difficulties, intellectual disability and mental health difficulties (e.g., requiring medication). Following completion of the experiment, participants were debriefed with an explanation about the purpose of the study. All participants were contacted via email one week after completion of the experiment to make sure there were not any undesired ‘after-effects’ from exposure to the VR system.

**Figure 1:**
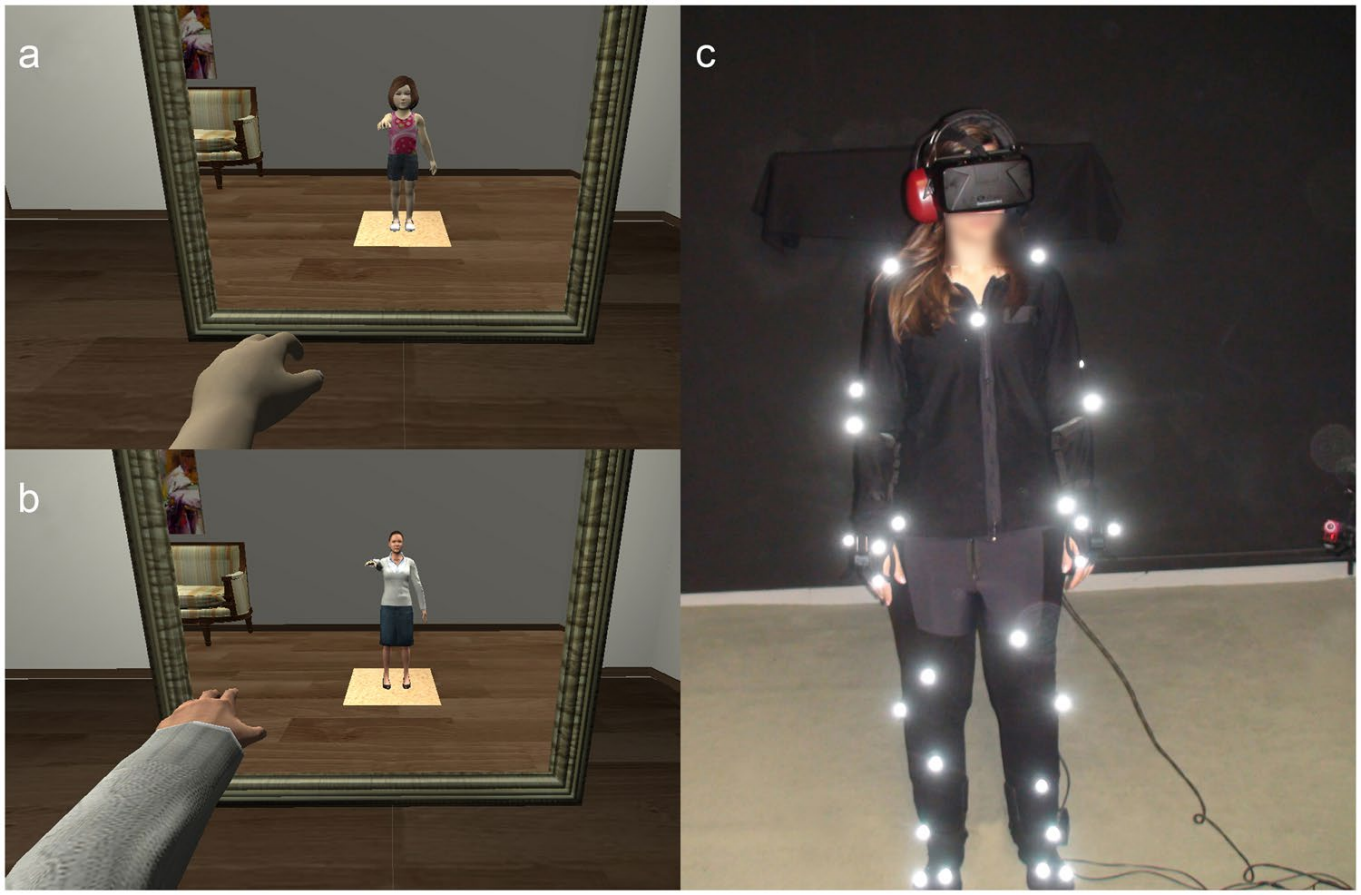
Experimental setup. The body of the participant was substituted by a gender-matched virtual body, viewed from 1PP, onto which body and head movements were mapped in real time. The body could also be seen as reflected in a virtual mirror, as shown. The body each participant viewed depended on the condition Child or Adult to which each one was assigned. (**a**) A female participant in a child’s body. (**b**) A female participant in a scaled-down adult’s body. (**c**) Participants were fitted with an Oculus Rift DK2 head-mounted display, and a headset Sennheiser HDA 200 fitting a microphone. Their body movements were tracked by 37 Optitrack markers. The virtual environment was implemented on the Unity3D platform (http://unity3d.com/unity) and animation-enabled models of female adult and children virtual bodies were purchased from Rocketbox Libraries (http://www.rocketbox-libraries.com/) and DAZ Studios (http://www.daz3d.com/), and were customized appropriately for the purposes of the study using 3D Studio Max 2014 academic version (http://www.autodesk.es/products/3ds-max/overview).

Informed consent for publication of identifying images or video footage (image or voice) was obtained from the people depicted in Fig. 1 and Supplementary Video S1.

### Participants

Thirty-four adult female healthy participants aged 40 to 68 years (mean age 55, SD = 7.64) with correct or corrected vision were recruited by advertisement and email around the campus of the University of Barcelona and the city of Barcelona. They had no prior knowledge of the experiment, and no or little prior experience of virtual reality. The experimental groups were comparable across a number of variables, including previous experience of VR, and time spent playing computer games (Table 1). Participants were compensated for their participation, by receiving 20€ (5€ after the end of the first phase, and the remaining 15€ after the end of the second phase).

**Table 1:** Experimental design - distribution of participants by condition, showing that the experimental groups were comparable across a number of variables.

Voice	Body	
	Adult-Child	Child-Adult
**Adult Voice**		
Female	n = 8	n = 9
Mean ± S.E Age	53.8 ± 2.8	57.2 ± 5.4
Median Code Previous VR Experience (IQR)	1(0)	1(0)
Median Code Games (IQR)	1.5(1)	1(0)
**Child Voice**		
Female	n = 8	n = 9
Mean ± S.E Age	53.3 ± 3.3	56.2 ± 2.2
Median Code Previous VR Experience (IQR)	1(0)	1(0)
Median Code Games (IQR)	1(0)	1(0)

^*^Groups Adult-Child and Child-Adult are formed based on the condition which each participant was assigned to during the first trial: child or adult body type. For each case the total number of participants, mean of ages, median and IQR values for participants’ experience in VR and hours of video games are given. Codes refer to a 1–7 Likert scale. For previous VR experience 1 means the least and 7 the most experience, and for hours spent playing video games 1 means least and 7 most.

### Experimental Design

The experiment was conducted as a mixed-groups counterbalanced design with two binary factors referred to as ‘Body’ and ‘Voice’. The ‘Body’ condition was a within-groups factor referring to whether participants experienced having the body of a 4-year-old child (condition Child) (Fig. 1a) or of an adult (condition Adult), which was scaled – down to match the height of the child body (91.5 cm) (Fig. 1b). The size of the virtual environment and proportions of the content were equivalent to their size and proportions in reality and identical in both conditions. The condition ‘Voice’ was designed as a between-groups factor, and refers to whether participants received real-time feedback of their own voice while speaking (Adult Voice), or a modified version of their voice that matched that of a child (Child Voice), which was dynamically processed by a real-time voice transformation system^47^.

Participants were randomly allocated to one of the four conditions, regarding whether they experienced the Adult Voice (own voice) or Child Voice feedback, and whether they first experienced a child virtual body and then an adult body, or an adult body first and then a child body. The experimental design can be seen in Table 1. The two trials were separated by 2 days.

### Procedures

Participants attended the experiment at pre-arranged times. Upon arriving, they were given an information sheet to read, and after they agreed to continue with the experiment, they were given a consent form to sign, and completed a demographics questionnaire. Before the VR exposure, participants were seated in front of a desktop computer fitted with the headset and were instructed to read out in a clear voice 9 target words displayed in sequence following the protocol described by Banakou and Slater^44^. Each word was recorded five times, in random order, using audio editing software, and was used as baseline data for later analysis. Then, they were fitted with the HMD, headset, and the body-tracking suit. This was followed by the actual VR exposure. Finally, participants were asked to complete an Implicit Association Test (IAT), which paired child or adult with self and other categories^26^, and a post-experience questionnaire. The whole procedure lasted approximately 45 minutes. Two female experimental operators were present throughout the whole experiment. All participants attended the second trial of the experiment two days after the first phase, and the procedures were identical to the ones presented above, except that the virtual body they experienced was the other one.

Further information is given in Supplementary Methods and Supplementary Video S1.

### Response Variables

#### Implicit Association Test (IAT)

The IAT was applied immediately after the virtual exposure on a desktop screen. The procedures were identical to those described by Banakou *et al*.^26^. During the first IAT block, the participant was asked to categorize visual stimuli into the two target categories, namely “Children” and “Adults”. The stimuli were pictures of adult and child faces appearing in the middle of the screen to be sorted into the appropriate category. In the *second* block, the participant was trained to press one key for ‘Me’ attributes and the other key on for ‘Others’ attributes. These attributes were presented as written words. They were personalized for each participant and corresponded to preferences and personal data, such as their names, ages, occupation, life status etc. These personal data and preferences had been obtained for each individual from the questionnaire administered before they started the experiment. The third and fourth blocks combined the target and the attribute discrimination that were subdivided into two blocks of 40 trials each. The subsequent *fifth* block reversed the target discrimination whilst the *sixth and seventh* blocks combined again the attribute and the previously reversed target discrimination. The order of combined blocks was counterbalanced between participants^48^.

#### Vocal Production Analysis

During the vocal production analysis, we extracted their FF across the 90 trials for each participant before (BaseF0) and after (F0) the exposure to the virtual environment to track the changes in the acoustics of the produced words (45 trials as baseline, and 45 trials after the voice modification)^44^. The computer software Praat^49^ was used for analysis of the speech and for reviewing trials from each participant for discontinuities caused by glottal fry.

#### Object Size Estimations

Participants were presented in random order with six virtual red colour cubes of different sizes over a period of 5 minutes^26^. All cubes were shown at 0.6 m away from the participant. The position from which participants looked at the objects was from a height of about 90 cm, equal to the height of the child and scaled adult virtual bodies. They were instructed to indicate the width of each cube by raising their hands and hold them straight in front of them, as if they would like to grasp it, and the size was measured as the distance between the palms.

#### Post-experience questionnaire

After each exposure a 21-statement post-questionnaire was administered to assess the subjective experience of participants (Table S1 in Supplementary Methods). A 7-point scale was used ranging from −3 to +3, with “0” indicating a neutral response on each question (with the scale varying from Strongly Disagree, −3, to Strongly Agree, +3). More specifically, these questions were related to the strength of body ownership (*VRBody*, *Mirror*) and agency (*Agency*) – here we require that the levels of body ownership and agency are the same between the two conditions – while others served as control questions (*VisualFeatures*, *TwoBodies*). There were also various questions relating to the experience of being a child (*Younger, Older, FeltChild*), and others regarding voice ownership and agency (*VRVoice, VoiceFeatures* and *VoiceAgency*). Three questions (*Surprise, Valence* and *Arousal*) were related to the emotional state of participants while being in the virtual environment. These were based on the Self-Assessment Manikin (SAM) introduced by Bradley *et al*.^50^.

### Data Availability

All data created during this research are openly available from the UK Data Service ReShare archive (https://dx.doi.org/10.5255/UKDA-SN-852815).

## Results

### Post-experience Questionnaire Responses

**Figure 2:**
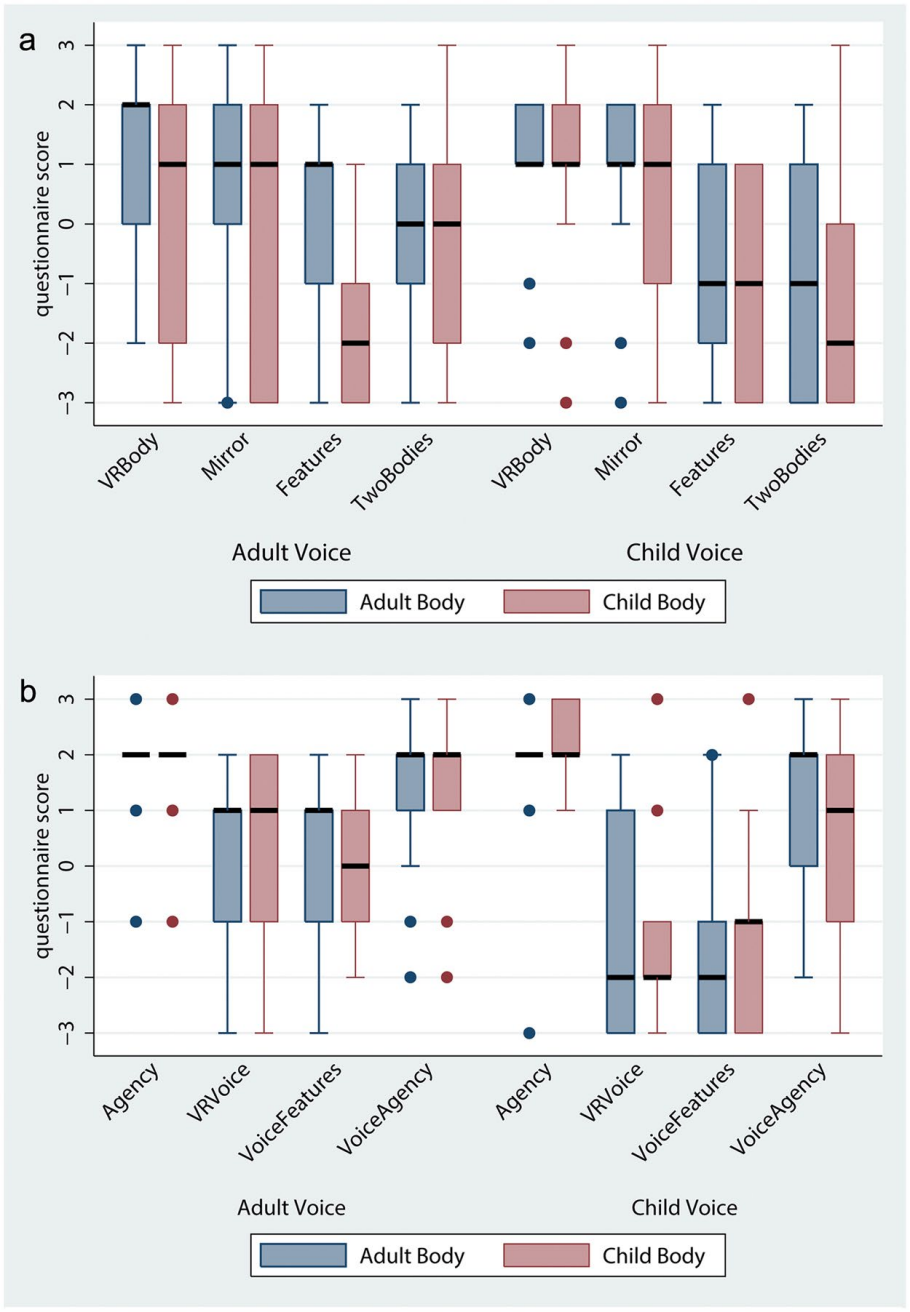
Box plots of questionnaires results (Table S1 in Supplementary Methods). Results are organized with respect to the two virtual bodies of conditions Child and Adult and with respect to the between-subjects factor Voice (Adult Voice versus Child Voice). The thicker horizontal lines are the medians and the boxes the interquartile ranges (IQR). The whiskers extend from max(min value, lower quartile − 1.5*IQR) to min(max value, upper quartile + 1.5*IQR). Points outside this range are shown individually.

The full set of questions and their scoring scales are presented in Table S1 in Supplementary Methods. First we consider the questionnaire responses on ownership and agency over the virtual body (Child versus Adult) and the feedback voice (Child versus Adult). Figure 2a shows the scores on body ownership. The variable *VRBody* refers to the degree to which participants felt as if the body they saw when looking towards themselves was their body, and *Mirror* refers to the body they saw in the mirror. *VisualFeatures* refers to the extent to which participants affirmed that the virtual body had physical features in common with themselves. *TwoBodies* is considered as a control question for VRBody and Mirror, and refers to the extent to which they felt they had two bodies.

It is clear that overall participants had the perceptual illusion of body ownership over the virtual body, irrespective of body type, with high scores for *VRBody*, and *Mirror*. The scores on these two across the conditions are very similar (the medians are all 1 or 2, and the interquartile ranges substantially overlap). However, participants tended to disagree with the feeling of having two bodies (*TwoBodies)* where the scores are generally much lower. Using the Wilcoxon matched pairs signed rank test comparing *VRBody* with *TwoBodies*, these were not significantly different only in the case of the Child Body and Adult Voice (P > 0.16), but in all other cases the differences were significant (Adult Body, Adult Voice: P = 0.020; Adult Body, Child Voice: P = 0.006; Child Body, Child Voice: P = 0.007). The results were similar comparing *Mirror* with *TwoBodies* - a non-significant difference for Child Body with Adult Voice (P > 0.47), with the remaining P-values 0.071, 0.004 and 0.018 respectively.

For *VisualFeatures*, as might be expected, there was a greater feeling of the virtual Adult body to have similar features to the participant’s body in the Adult Voice (own voice) than in the Child Voice condition. A mixed model ordered logistic regression of *VisualFeatures* on the Body and Voice factors reveals a strong effect of the Child Body, which reduces the extent to which participants reported the virtual body as sharing features in common with their own (z = −3.26, P = 0.001).

Figure 2b shows the results for *Agency*, which refers to the extent to which participants affirmed that the virtual body’s movements were their own. *Agency* had a median of 2 in all conditions, with small IQRs, reflecting the fact that the real-time motion-capture system worked well, and the virtual body moved in synchrony with real body movements. Figure 2b also shows the results for voice ownership and agency over the speaking (*VRVoice* and *VoiceAgency*). For *VRVoice*, referring to the extent to which participants felt the voice they heard while speaking was theirs, an ordered logistic regression over the factors Body and Voice and their interaction did not show significant interaction (P = 0.734); however, removing the interaction term resulted in a significant effect of Voice (z = −2.08; P = 0.038), with Body not significant. Hence ownership over the voice was reduced in the Child Voice condition.

Regarding the acoustical features of the heard voice (*VoiceFeatures)*, the analysis showed a significant main effect of Voice (z = −2.21; P = 0.027), while the main effect of Body (P = 0.797) and the interaction effect between the two factors (P = 0.863) were not significant. Removing the interaction term resulted in a similar significant effect of Voice (z = −2.36; P = 0.018), with Body not significant. Overall, participants felt that the voice they heard when they spoke was more their own voice and resembled more the acoustical features of their own voice in the Adult Voice (own voice) than in the Child Voice condition; these ratings did not interact with the Body condition. Nevertheless, ratings for *VoiceAgency* were high for all conditions, as shown in Fig. 2b, and there were no significant main effects or interaction effect between the two factors.

**Figure 3:**
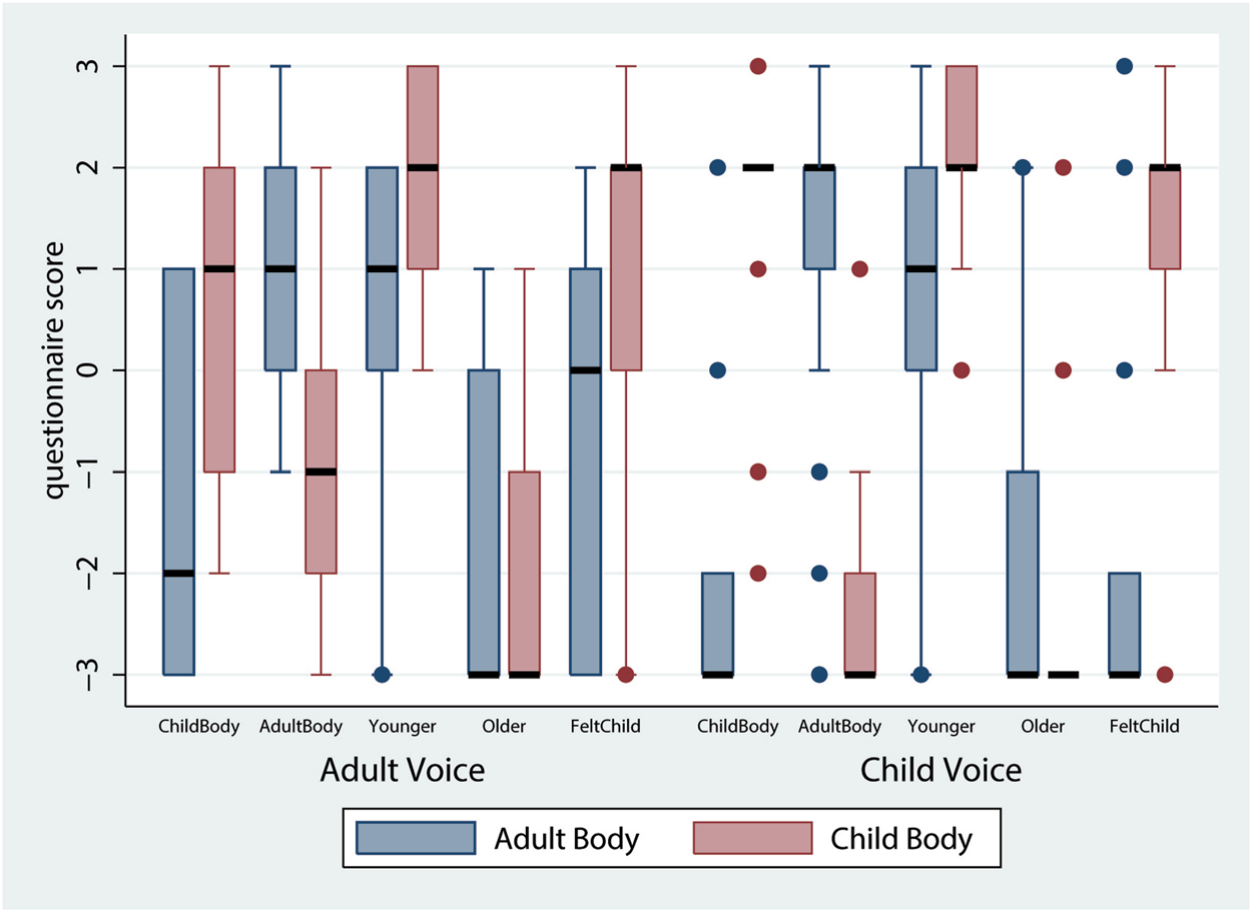
Box plots of questionnaires results related to the felt age of the actual body. Results are organized with respect to the two virtual bodies of conditions Child and Adult and with respect to the between-subjects factor Voice (Adult Voice versus Child Voice). The thicker horizontal lines are the medians and the boxes the interquartile ranges.

Regarding the questions related to the extent to which participants felt younger, older or like a child (*Younger*, *Older*, *FeltChild*), ordered logistic regressions over the Body and Voice factors showed a significant main effect of Body for *Younger* (z = 1.96; P = 0.049) and *FeltChild* (z = 2.75; P = 0.006) scores, while there was no main effect of Voice or interaction between the two factors. Removing the interaction term resulted in similar significant main effect of Body for *Younger* (z = 3.63; P = 0.000) and *FeltChild* (z = 4.33; P = 0.000), with *Voice* not significant. As it can be seen in Fig. 3 participants reported feeling younger, in the Child Body condition. There were no significant main effects or interaction related to participants reports of feeling older (*Older*). Overall, participants felt younger as a child but not older as an adult.

Regarding the emotional state of participants, ordered logistic regressions over the factors Body and Voice showed a significant interaction effect for *Valence* (z = 2.44; P = 0.015) where Child Voice and Child body together are associated with greater values of Valence. While all the scores are high, they are highest just in the case of the Child Body and Child Voice (Figure S1 in Supplementary Methods). There were no differences with respect to *Arousal* or *Surprise*.

### Object size estimations

**Figure 4:**
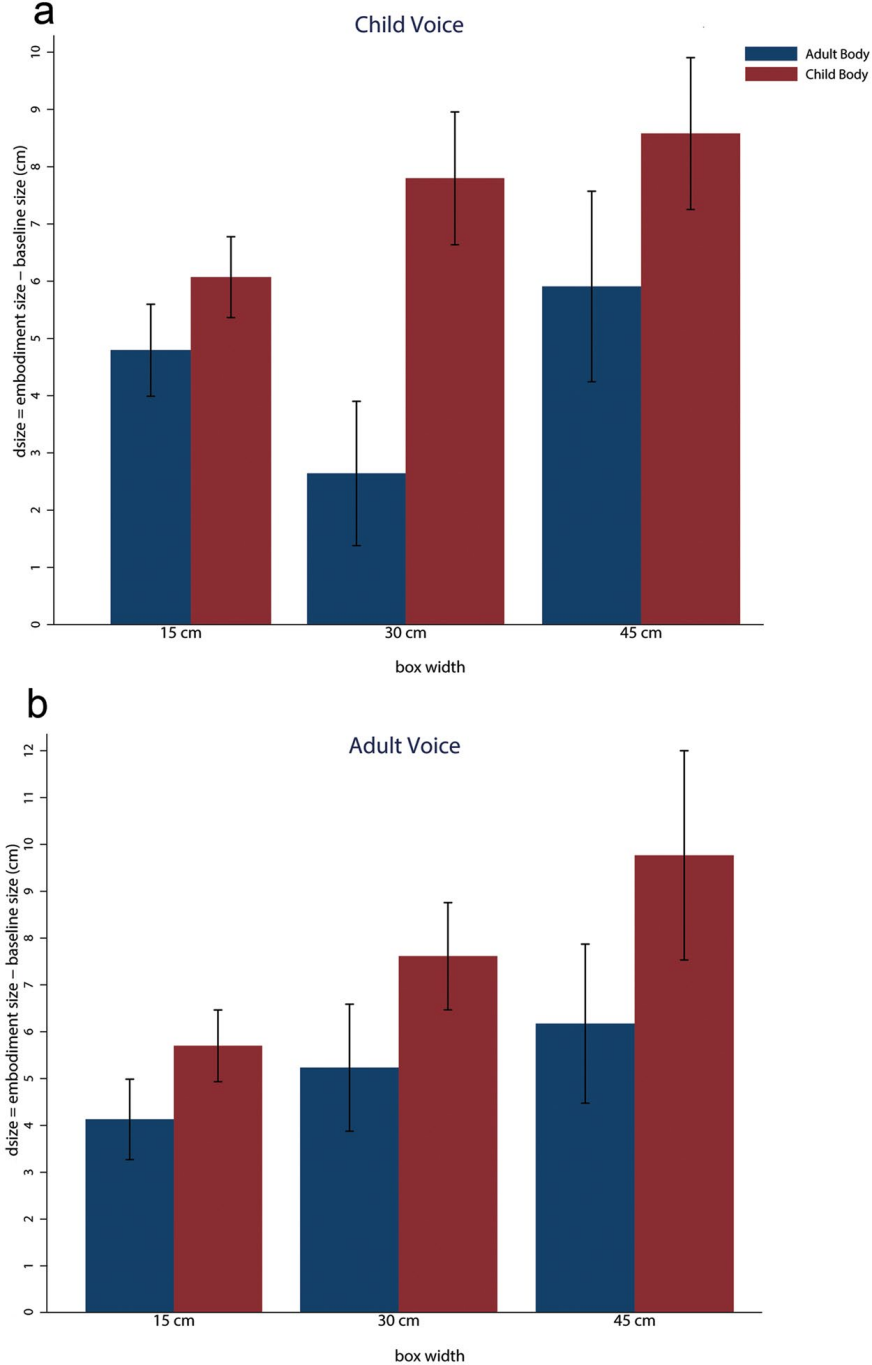
Bar chart size-estimation results. The heights are means and the bars SEMs. The variable *dmean15*, *dmean30*, and *dmean45* are the differences between the post-embodiment size estimations and pre-embodiment (baseline) size estimations, for the boxes of the three different sizes.

Due to the relationship between the perception of one’s own body size and the perception of sizes of external objects, we were interested in the effects of Body and Voice conditions on participants’ estimations of the size of the cubes they were presented with before and after each experimental session. For each of the three differently sized cubes (15 cm, 30 cm, and 45 cm) we took the mean of the five estimates participants provided before each experimental session (baseline), and the mean of the fifteen estimates (five estimates in three different orientations) provided after each experimental session and calculated the difference between the two means (dsize)^26^. We carried out mixed-effects regression analyses for the variables dsize15, dsize30 and dsize45. The results, as illustrated in Fig. 4, showed a significant main effect of Body for dsize15 (P = 0.048), dsize30 (P < 0.0005) and dsize45 (P = 0.022), while there was no main effect of Voice or interaction between the two factors. Overall participants estimated the objects as being larger during the Child Body condition as compared to the Adult Body condition. Moreover, in each case, as it can be seen from Fig. 4, there is always overestimation of size irrespective of whether it is the Adult or Child body (the corresponding significance levels for this are P < 0.0005 for each case). The results showing larger object overestimation during the Child Body condition as compared to the Adult Body condition conforms with the findings reported by van der Hoort and colleagues^27^ that embodiment in a small body leads to overestimation of object sizes. Both of these results – the effect of the small body and the greater overestimation of size in the case of the Child compared to the Adult body - replicate our previous findings^27^.

### Vocal Production Analyses

**Figure 5:**
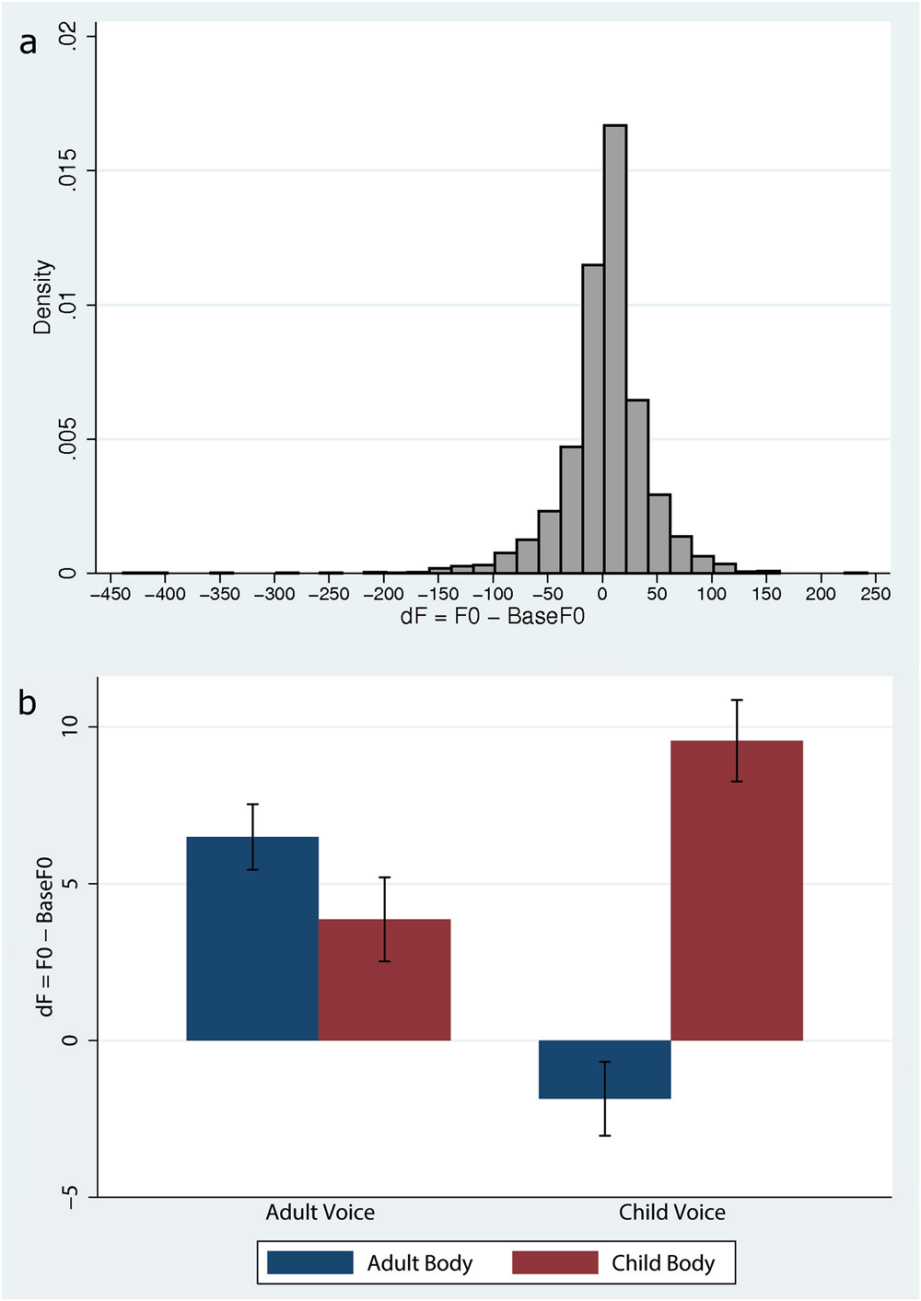
Vocal production analysis: (**a**) histogram of dF over all observations, (**b**) Mean and SE of dF (collapsed) by Voice and Body with outliers removed.

None of the participants showed glottal fry that would have required them to be excluded from the data. For each participant we extracted the fundamental frequency (F0) of all trials in order to track the changes in the acoustics of the produced words^44^. We then calculated the mean F0 before and after the exposure to the virtual environment (45 trials before – BaseF0, and 45 after the exposure - F0). The variable of interest is dF = F0–BaseF0. Figure 5a shows the histogram of dF over all observations. It is clear that there are some extreme outliers (e.g., the mean dF = 2.9, SD = 40.57, but there were values such as −438 and 242). We eliminated outliers by removing all observations outside of the mean ± 3*standard deviations. As a result, 1.5% of the total number of observations were excluded from the subsequent analysis. Figure 5b shows an interaction effect, with the greatest change in dF in the Child Body with the Child Voice. We carried out a mixed regression analysis with dF as the dependent variable, Body and Voice as the fixed effects variables, and with random effects over subject id. We also allowed for differences in the effect of the different words. This results in a significant main effect for Voice (z = −3.65, P < 0.0005) and a significant interaction effect (z = 6.42, P < 0.0005) highlighting what is shown in Fig. 5b that the effect of Body is different between the Adult Voice and Child Voice.

We compare all marginal contrasts at the overall level of 5% significance (Scheffe’s method). Tests for significance are summarized in Table 2, where significant differences are indicated (i.e., the 95% confidence intervals of left hand expression - right hand expression do not include 0). We conclude that the Child Body condition resulted in a higher dF than the Adult Body condition, but more important are the interaction terms: For those participants in the Child Voice condition, the Child Body resulted in greater dF than the Adult Body. Further, in the Adult Body condition the Child Voice condition resulted in smaller dF than the Adult Voice condition. This can be explained as an overcompensation from participants when there were inconsistencies between the voice feedback and the body they saw^44^.

**Table 2:** Summary of comparisons in vocal production analyses (the 95% confidence intervals of left hand expression - right hand expression do not include 0).

	95% CI for left hand side expression - right hand side expression	
**dF(Child Body) > dF(Adult Body)**	**2.1**	**6.5**
dF(Child Voice)~dF(Adult Voice)	−5.6	2.5
**dF(AdultBody#ChildVoice) < dF(AdultBody#AdultVoice)**	**−15.1**	**−2.0**
dF(ChildBody#AdultVoice)~dF(AdultBody#AdultVoice)	−6.9	1.7
dF(ChildBody#ChildVoice)~dF(AdultBody#AdultVoice)	−3.7	9.4
dF(ChildBody#AdultVoice)~dF(AdultBody#ChildVoice)	−0.7	12.4
**dF(ChildBody#ChildVoice) > dF(AdultBody#ChildVoice)**	**7.0**	**15.7**
dF(ChildBody#ChildVoice)~dF(ChildBody#AdultVoice)	−1.1	12.0

Comparisons showing significant differences between conditions are displayed in bold font, while the rest of the comparisons are displayed in normal font.

### Implicit Association Test Scores

**Figure 6:**
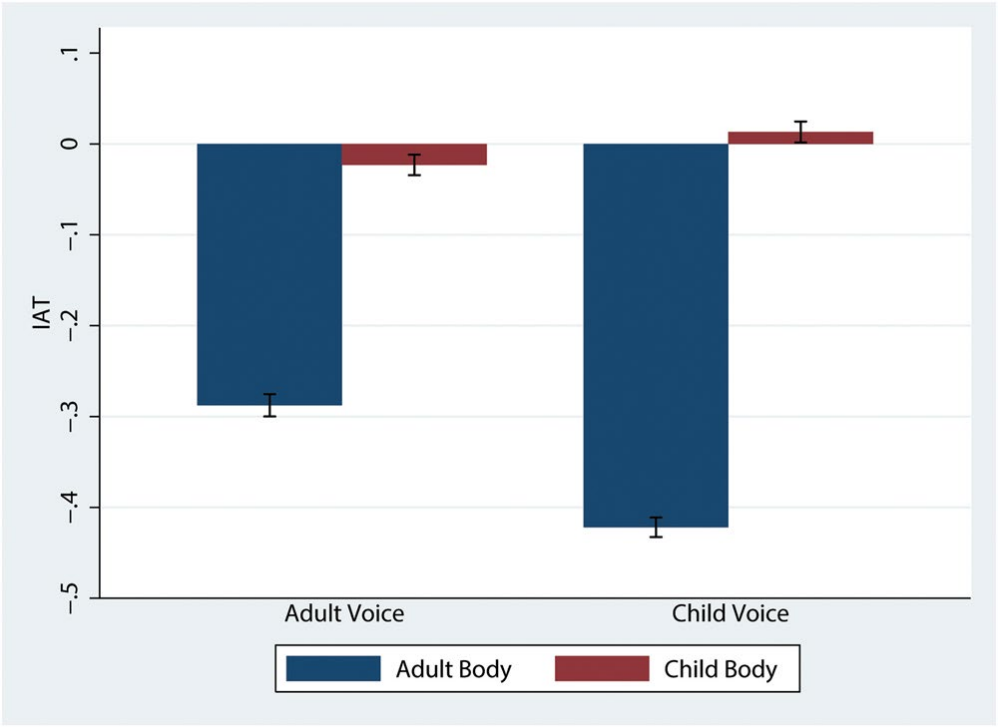
Bar chart representing the Mean and SE of IAT scores by Voice and Body. More positive scores reflect stronger associations for Me-Child relatively to Me-Adult.

Participants also completed an IAT at the end of the different experimental conditions. This IAT paired ‘child’ or ‘adult’ with the categories of ‘self’ and ‘other’. In the interpretation of the IAT scores, more positive scores reflect stronger associations for Me-Child relatively to Me-Adult. Figure 6 shows that the Child Body is associated with a greater IAT. A mixed effects regression shows a significant effect of Body (z = 2.94; P = 0.003) and no significant effect of Voice (P = 0.220) and no interaction between the two factors (P = 0.181). The result is due to the fact that in the Child Body conditions the IAT is around 0, but in the Adult Body conditions the IAT is negative. This means that while in the Adult Body condition participants had a strong Me-Adult association, this strong association disappeared in the Child Body condition. Results of the IAT test are consistent with all the other results, showing that the factor Body has a large overall effect, but Voice only in the case of the fundamental frequency analysis.

## Discussion

The present study replicates previous findings, showing that it is possible to generate a subjective illusion of ownership with respect to a virtual body that represents either a child or a scaled-down adult even though our participants were adults over 40 years old (compared to younger students in previous studies). In this study, apart from manipulating the visual aspects of the observed virtual body, we also manipulated acoustic cues of speech that related to one’s body age. Considering the first issue as to whether the strength of the illusion differs for each of the auditory feedback cues, we found that auditory cues signalling having a child’s voice did not seem to have an effect on strengthening the feelings of ownership or agency over the virtual child body. However, the results suggest that incongruent auditory cues (a) reduced the feeling of similarity between the features of the virtual adult body and those of the participant’s real body, and (b) affected participants’ own vocal production in line with^44^. Moreover, previous findings^44^ are extended because we found here that the direction of the adaptation depended on whether the heard child voice was congruent or incongruent with the age of the virtual body. In relation to the second issue, whether adults may come to experience a child-like voice as their own voice when embodying a child body - representation, we found that agency over the actions of the body and the voice was high in all conditions. In relation to the other three issues we considered (i.e., influences on perception of sizes, child-like vs. adult-like self-associations, feelings of being younger and emotional state), we found that embodying the virtual child body made our participants estimate the objects as being larger, replicating previous findings^26^. With respect to the IAT we found that the Me-Adult association vanished with embodiment of the virtual adult body, consistent with^26^, and also made participants feel younger. Finally, for those participants experiencing a child-like voice, embodying the virtual child body made our participants feel happier than when they saw themselves in an adult body. We discuss the observed effects, implications and topics for future research in further detail below.

With respect to the main aim of the study we showed that the illusion of owning a child’s body could be successfully induced even in older adults. This finding expands on earlier results indicating that it is straightforward to generate such body illusions in VR with different body forms, including that of a child^16,24,26,51,52^. Further, our results showed no significant differences with respect to body ownership between the different body and voice conditions, with overall high scores in all cases. Similarly, agency over the actions of the body and the voice is high in all conditions. This result serves as a reference point, showing that there are no variations in the extent of the illusion of ownership between the various conditions that could account for any other findings. Notably, there was an interesting difference in the subjective report of physical resemblance between the participants and the scaled-down adult virtual body in the Adult Voice (own voice) condition, something that does not hold for the Child voice one. It could be argued that employing incongruence between visual and audio feedback reduced the subjective feeling of resemblance with the adult virtual body; this in turn, could be interpreted as a reduction in the feeling of being an adult or as a lack of ownership over the adult virtual body. Research on multisensory perception has suggested a number of general principles for crossmodal integration of information received across the different senses, including the need for keeping incongruences across the senses under a certain threshold^53–55^. At the same time, voice seems to influence the subjective feeling of being a child, as the difference between the Adult and Child Body conditions was much greater in the Child Voice than in the Adult Voice condition. Similarly, participants reported feeling younger when embodied in the child virtual body.

The effect of the voice on the experience of having a child’s body is also evident from the results of the manipulation of participants’ voice feedback. Past studies have provided behavioral^56–58^ and neuroimaging^59,60^ evidence for the role of auditory feedback both in articulatory and vocal motor control, but also for the recognition of one’s own voice^61^. Such feedback perturbation paradigms have shown that people tend to compensate for a change of the fundamental frequncy F0 in online auditory feedback during vocal production, either by shifting their F0 in the direction of the feedback signal^56,61^, or in the opposite direction. Specifically, it is argued that shifting towards the stimulus voice serves to bring the participant’s voice to agree with that of the external source. On the contrary, compensating works as an error correction mechanism to return the signal closer to that intended by the speaker. In our study, for those participants embodied in the child virtual body, and with a child voice feedback, there was an increase in the F0, whereas for those in the adult body there was a shift towards the opposite direction. It is thus evident that congruency between auditory and visual feedback caused greater changes regarding voice recalibration towards the signal voice. Although there also seems to be an increase for those participants in the child body but with the own voice, this change is smaller.

Furthermore, we report differences in object size estimations with respect to the body form. We show that the Child body always had the effect of increasing the mean size estimation over the baseline compared to the Adult body. These results replicate earlier work, where it was shown that when adult participants were embodied in a child virtual body there was a much greater overestimation of sizes compared to when they were embodied in a scaled-down adult virtual body^26^. Our findings provide additional evidence to the earlier notion that, as well as perception about one’s body size serving a reference for the external world^62–64^, higher-level cognitive processes (i.e. age perception) can also influence our perceptual interpretation of sizes. Unlike the body form, the voice factor did not have any significant effect on these results. It has been previously shown that altering other sounds produced by one’s body, such as altering the perceived position of the sounds produced by one’s hand when tapping on a surface, can result in recalibration of the represented length of one’s arm and that this in turn can lead to changes in the perceived size of external objects in contact with one’s arm^28,29^. These findings suggested that tactile perception is referenced to an implicit body-representation which is updated through auditory feedback, presumably by auditory-induced recalibration of receptive fields in primary somatosensory cortex, as observed also for some cases of visual bodily feedback^28,65^. Nevertheless, as suggested before, sensory information about one’s body and about objects is used differently in distinct tasks and there might be other top-down factors (e.g. contextual/task demands) that might influence object size judgments^66^. In light of this suggestion, and given our current results, it is not possible to tell whether manipulation of voice had an effect of the represented body size or not, as it is possible that the task chosen, estimation of size of observed objects, was not able to capture these changes.

In addition, we provide evidence on how an altered body - representation can affect perception about one’s identification and subsequent implicit attitudes and behaviors. This result is in line with earlier work^26^, showing the impact of body form on self- and other-categorization. Specifically, those embodied in the child virtual body responded faster with respect to classification of the self as a child, with overall greater IAT scores. On the contrary, those in the scaled-down adult condition showed more negative scores, thus being faster at self-categorization with adult-like attributes. A recent review of a number of experimental studies suggests that body ownership illusions can lead to changes in social cognition as a function of the type of body - representation^3^. For example, previous research investigated positive and negative associations with an embodied social group^22,24,25,67^. The present work, together with previous work^26^, demonstrates the significant role of self - association in attitudinal changes.

Finally, our results showed the positive impact that the illusion of having a child’s body has in feelings of being younger and in emotional state, as our participants reported feeling happier after embodying the child’s body. Previous studies have shown the impact that body-represntation can have on emotional state and self-esteem^31,68^. This finding opens up possibilities for applications in health and rehabilitations contexts aiming to increase confidence about one’s body, and potentially, provide alternatives to medication for treatments and therapies in clinical cases.

## Electronic supplementary material

Supplementary Video

Supplementary Information
