# Author Correction: Embodiment in a Child-Like Talking Virtual Body Influences Object Size Perception, Self-Identification, and Subsequent Real Speaking

**DOI:** 10.1038/s41598-018-23231-7

**Published:** 2018-03-14

**Authors:** Ana Tajadura-Jiménez, Domna Banakou, Nadia Bianchi-Berthouze, Mel Slater

**Affiliations:** 1 UCL Interaction Centre (UCLIC), University College London, London, UK; 2 Universidad Loyola Andalucía, Department of Psychology, Seville, Spain; 3 Universidad Loyola Andalucía, Human Neuroscience Lab, Seville, Spain; 4 Event Lab, Department of Clinical Psychology and Psychobiology, Faculty of Psychology, Barcelona, Spain; 5 Institute of Neurosciences, University of Barcelona, Barcelona, Spain; 6 Institució Catalana de Recerca i Estudis Avançats (ICREA), Barcelona, Spain; 7 Department of Computer Science, University College London, London, UK

Correction to: *Scientific Reports* 10.1038/s41598-017-09497-3, published online 29 August 2017

The Acknowledgements section in this Article is incomplete.

“ATJ was supported by the ESRC grant ES/K001477/1 (“The hearing body”) and by a Spanish “Ministerio de Economía y Competitividad” Ramón y Cajal research contract (RYC-2014–15421). DB was supported by PSI2014-56301-R Ser Einstein: La Influencia de Internalizar un Cuerpo Virtual en la Inteligencia, Ministerio de Economía, Industria y Competitividad of Spain. This work was also supported by the Virtual Embodiment and Robotic Re-Embodiment (VERE) Integrated Project funded under the European Seventh Framework Programme, Future and Emerging Technologies, Grant Agreement 257695. We thank Torsten Marquardt for his help with the experimental design, Professor Mark Huckvale for his help with the design of the the auditory stimuli and for provinding the real-time voice-transformation system and Andrea Yeung for her assistance with the vocal production analysis.”

should read:

“ATJ was supported by the ESRC grant ES/K001477/1 (“The hearing body”) and by RYC-2014–15421 and PSI2016-79004-R (“MAGIC SHOES: Changing sedentary lifestyles by altering mental body-representation using sensory feedback”; AEI/FEDER, UE), Ministerio de Economía, Industria y Competitividad of Spain. DB was supported by PSI2014-56301-R Ser Einstein: La Influencia de Internalizar un Cuerpo Virtual en la Inteligencia, Ministerio de Economía, Industria y Competitividad of Spain. This work was also supported by the Virtual Embodiment and Robotic Re-Embodiment (VERE) Integrated Project funded under the European Seventh Framework Programme, Future and Emerging Technologies, Grant Agreement 257695. We thank Torsten Marquardt for his help with the experimental design, Professor Mark Huckvale for his help with the design of the auditory stimuli and for providing the real-time voice-transformation system and Andrea Yeung for her assistance with the vocal production analysis.”

