# Supplementary material for: Embodiment in a Child-Like Talking Virtual Body Influences Object Size Perception, Self-Identification, and Subsequent Real Speaking
Source: Sci Rep. 2017 Aug 29;7:9637. doi: 10.1038/s41598-017-09497-3 (PMC5575082; doi:10.1038/s41598-017-09497-3)
Supplement: Supplementary file 2 — Supplementary Information [file 41598_2017_9497_MOESM2_ESM.doc]

**Embodiment in a Child-Like Talking Virtual Body Influences Object Size Perception, Self-Identification, and Subsequent Real Speaking**

Ana Tajadura-Jiménez*1, 2, 3[[1]](#footnote-2)#, Domna Banakou4, 5[[2]](#footnote-3)#, Nadia Bianchi-Berthouze^1^, Mel Slater^*4, 5, 6, 7^

^1^ UCL Interaction Centre (UCLIC), University College London, London, UK

^2^ Universidad Loyola Andalucía, Department of Psychology, Seville, Spain

^3^ Universidad Loyola Andalucía, Human Neuroscience Lab, Seville, Spain

^4^ Event Lab, Department of Clinical Psychology and Psychobiology, Faculty of Psychology, Barcelona, Spain

^5^ Institute of Neurosciences, University of Barcelona, 08035 Barcelona, Spain

^6^ Institució Catalana de Recerca i Estudis Avançats (ICREA), Passeig Lluís Companys, 23 08010 Barcelona, Spain

^7^ Department of Computer Science, University College London, London WC1E 6BT, United Kingdom.

*Correspondence:

Ana Tajadura-Jiménez: [a.tajadura@ucl.ac.uk](mailto:a.tajadura@ucl.ac.uk)

Mel Slater: melslater@ub.edu

**SI Materials and Methods**

1. Procedures

**Scenario.** The position of all participants was controlled through Velcro strips on the floor which were used to mark where they should stand during the experiment. These positions corresponded to the centre of the physical and virtual room. Participants were instructed to turn and move their heads and bodies but not walk more than two steps away from that area unless instructed otherwise. That area was represented in the virtual environment by a virtual mat, on which participants were asked to stand.

During the first part of the experiment, participants entered a virtual outdoor scene where they trained their object-size estimation capability thereby also familiarizing themselves with the task of estimating sizes of objects in VR. In this setup they had no virtual body. During this task they were presented in random order with six virtual red colour cubes of different sizes (15, 25, 30, 45, 60, and 75 cm) in front of them over a period of 5 minutes. All cubes were shown in the same position and at 0.6 m away from the participant. The position from which participants looked at the objects was from a height of about 90 cm equal to the height of the child and scaled adult avatars. Participants were instructed to indicate the width of each cube by raising their hands and hold them straight in front of them, as if they would like to grasp it, and the size was measured as the distance between the palms. The distance was calculated using the tracking devices on their hands and was automatically recorded for each object separately. An offset corresponding to the distance between the tracking device and the participant’s palms individually was also taken into account when estimating the final results (the average among all participants was 8cm). After each size estimation participants were given visual feedback in the form of words on the screen regarding their measurements, which categorized their estimations as “Too Big”, “Too Small” or “Correct”. In cases where measurements were other than “Correct” they were instructed to relax their arms and try again, until they achieved a “Correct” feedback. Only then was the next virtual object presented to them. Each measurement was classified with a ±4 cm tolerance (e. g for 15cm virtual objects “Correct” estimation varied from 11cm to 19 cm).

Next, participants removed the HMD, and they were asked to complete a personal traits questionnaire the information from which was used later during the IAT. For example, they had to provide their age, gender, profession etc. Immediately after completing the questionnaire they put on the HMD again and the second and main part of the experiment started. Participants entered the same training virtual scene, and still with no virtual body. They were asked to repeat the object-size estimation task. Red coloured cubes of 15 cm, 30 cm, and 45 cm were each presented five times in random order. Each virtual object remained visible in front of participants at a constant distance (of 0.6 cm) for 5 seconds. After each cube disappeared they were asked to indicate their estimate by the distance between their hands, and the measurements were recorded with the same procedure as described before, but without any feedback as to the correctness of the size estimations. This provided the baseline size estimations.

While participants still wore the HMD a new scene was loaded. This portrayed a virtual living room that included a virtual mirror. The body of the participant was substituted by a gender-matched virtual body, seen from a first person perspective. The participant’s head and body movements were mapped in real time to the virtual body. They could see this body both by looking directly towards their real body, and also in the virtual mirror. The body seen by each participant depended on the condition Adult or Child. The feedback of their voice also depended on the condition Adult voice or Child Voice. Following the procedures described in ^1-4^, a series of tasks were then assigned to the participants; first they were asked to perform a simple set of stretching exercises that were communicated to them via audio, in order to explore the capabilities and real time motion of the virtual body, including movements of their arms, legs and feet. They were asked to continue performing these exercises by themselves and also look around the virtual room in all directions. During this visual exploration, participants were asked to state and describe what they saw, in order to make sure that they were paying attention and the system worked properly. These tasks allowed participants to familiarize themselves with their virtual body and voice and their environment. After the exploration period (5 minutes), they were asked to repeat the size estimation task. Each object was measured five times in random order at three different locations all at the same distance from the participant equal to that of the control measurements. The heights at which each object was placed were always the same, and were the same as in the control condition. After each object appeared the screen went black and only then were participants allowed to estimate the sizes, so that they did not have visual feedback of their virtual arms and hands. After the size estimation task, the HMD displayed a black background with written instructions prompting them to read out loud the nine target words that then appeared in front of them in random order. Each word was recorded 5 times.

Finally, after removing the HMD, they completed the IAT on a desktop screen, and then filled in the post-experimental questionnaire. Next, they were paid and debriefed. The whole procedure lasted approximately 45 minutes. The experimental operators (female) were present throughout the whole experiment.

All participants attended the second trial of the experiment two days after the first phase, and the procedures were identical to the ones presented above, except that the avatar body they experienced was the other one.

1. Materials

**Environment**. The experiment was conducted in a VR lab (width: 2.96 m, length: 3.4 m - back wall to curtain - height: 2.87 m). Participants were fitted with an Oculus Rift DK2 (<https://www.oculus.com/en-us/dk2/>) head-mounted display (HMD) (Fig. 1C). This has a nominal field-of-view 100°, with a resolution of 960×1080 per eye displayed at 60Hz. Participants were also required to wear an Optitrack full body motion capture suit that uses 37 markers and calculated with the Motive software (<https://www.naturalpoint.com/optitrack/products/motive>) to track their movements (Fig. 1C). The infrared technology was implemented with a 12-camera truss setup by OptiTrack. The virtual environment was implemented on the Unity3D platform (<http://unity3d.com/unity>) and animation-enabled models of female adult and children virtual bodies were purchased from Rocketbox Libraries (<http://www.rocketbox-libraries.com/>) and DAZ Studios (<http://www.daz3d.com/>), and were customized appropriately for the purposes of the study using 3D Studio Max 2014 academic version (<http://www.autodesk.es/products/3ds-max/overview>).

**Audio Specifications.** A real-time voice-transformation system^5^ was used to dynamically modify the characteristics of the participants’ speech. The speech signal was picked up by a microphone (Core Sound, frequency response 20 Hz-20 kHz) mounted onto a headset that participants wore. The signal was processed on a laptop computer running the voice transformation system in one of two modes: “Child voice” or “Adult voice”. In the child voice mode, the sound of the participant’s voice was modified by scaling the effective vocal tract length by a factor of 0.8 and by scaling the voice pitch upwards by 4 semitones. Note that there are significant correlations between body size and vocal tract resonance frequencies as well as between larynx size and voice fundamental frequency in speakers. The applied voice pitch scaling and 20% increase in vocal tract resonance frequencies of our adult female speakers resulted in speech sounds that are consistent with those produced by girls at age 4^6-9^. In the adult voice mode, no tract size or pitch scaling were applied so that the sound of participant’s voice was unmodified. The voice transformation system introduced a delay of approximately 50 ms in the processed signal – note that this delay was similar in both child voice and adult voice modes. The transformed sound was fed back to participants via closed headphones (Sennheiser HDA 200) with high passive ambient noise attenuation (>35 dBA) that muffled the actual sound of their own voice that participants would normally get by air-conduction through their ears (although note that approximately 50% of the sound energy when hearing one’s own voice is transmitted through bone conduction^10, 11^.

1. Analysis

**Statistical Methods.** All results were obtained with Stata 14 ([www.stata.com](http://www.stata.com/)). Each of the questionnaire scores was analyzed with ordinal logistic regression (the ologit Stata function) on the factors Body and Voice. This method was used rather than non-parametric methods because these do not allow exploring interaction effects in mixed designs, whereas interaction effects are a standard part of logistic models. Moreover, it is not appropriate to use standard analyses of variance (ANOVA) on ordinal data because these data violate several assumptions of the general linear model. Object-size estimates, vocal production data and IAT data were analyzed using a mixed-effects (fixed- and random-effects) ANOVA model.


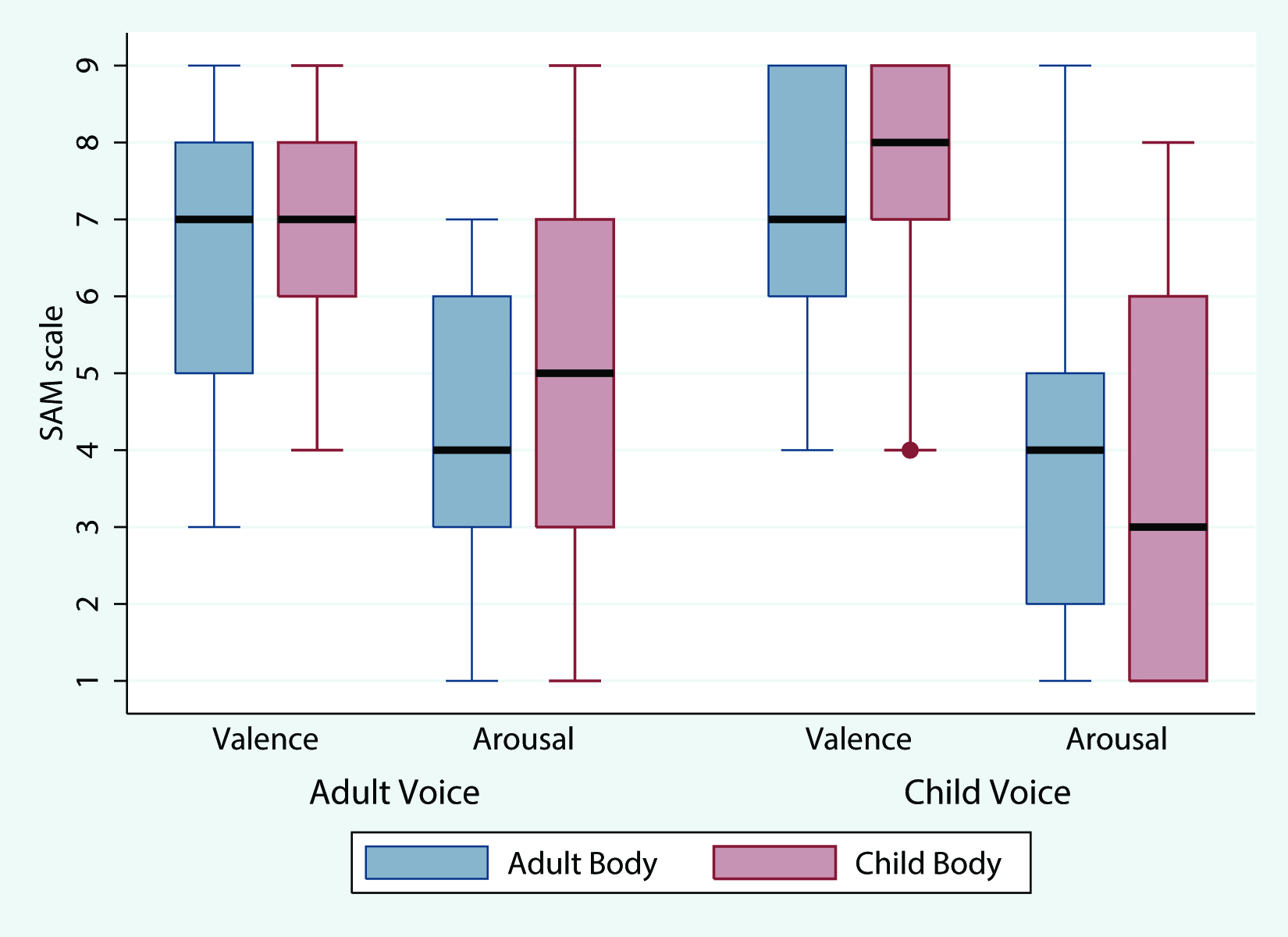


**Figure S1.** Box plots of questionnaires results of the Self-Assessment Manikin (SAM) used to rate the affective dimensions of valence and arousal.

**Table S1**. Post-Experience Questionnaire

| **Question ID** | **Variable Name** | **Question** | **Scoring Scale** |
| --- | --- | --- | --- |
| **Q1** | VRBody | ‘How much did you feel that the *virtual body* you saw when looking down at yourself was your own body?’ | Not at all/ Very much(-3…3) |
| **Q2** | Mirror | ‘How much did you feel that the *virtual body* you saw when looking at yourself in the *mirror* was your own body?’ | Not at all/ Very much(-3…3) |
| **Q3** | VisualFeatures | ‘How much did you feel that your virtual body resembled your own (real) body in terms of shape, skin tone or other visual features?’ | Not at all/ Very much(-3…3) |
| **Q4** | TwoBodies | ‘How much did you feel as if you had two bodies?’ | Not at all/ Very much(-3…3) |
| **Q5** | Agency | ‘How much did you feel that the movements of the virtual body were caused by your own movements? | Not at all/ Very much(-3…3) |
| **Q6** | RoomSize | ‘Did you feel that the virtual environment compared to your everyday environment was: ’ | Smaller/ Bigger  (-3…3) |
| **Q7** | VBSize (VB=VirtualBody) | ‘Did you feel that your entire virtual body as compared to your real body was’ | Smaller/ Bigger  (-3…3) |
| **Q8** | VBSize(torso) | ‘Did you feel that the torso of your virtual body as compared to the trunk of your real body was’ | Smaller/ Bigger  (-3…3) |
| **Q9** | VBSize(legs) | ‘Did you feel that the legs of your virtual body as compared to the legs of your real body were’ | Shorter/ Longer  (-3…3) |
| **Q10** | RBSize (RB=RealBody) | ‘While being in the virtual environment, did you feel your unseen real body’ | Smaller/ Bigger  (-3…3) |
| **Q11** | VRVoice | ‘How much did you feel that the voice you heard when you spoke was your own voice?’ | Not at all/ Very much(-3…3) |
| **Q12** | VoiceFeatures | ‘How much did you feel that the voice you heard when you spoke resembled your (real) voice in terms of tone, pitch or other acoustical features?’ | Not at all/ Very much(-3…3) |
| **Q13** | VoiceAgency | ‘How much did you feel that you caused the voice you heard?’ | Not at all/ Very much(-3…3) |
| **Q14** | Surprise | ‘While being in the virtual room, were the feelings about your real body surprising and unexpected?’ | Not at all/ Very much(-3…3) |
| **Q15** | Valence  Arousal | Please circle the manikin that you think better expresses how you felt while being in the virtual room (Self-assessment manikin; Bradley and Lang, 1994). | Unhappy Annoyed Negative/ Happy Pleased Positive  Relaxed  Calm  Dull/ Stimulated Excited Jittery |
| **Q16** | VBAge | ‘Of what age was the virtual body you had during the experience?’ | 3-7 years old  8-12 years old  13-17 years old  18-30 years old  > 30 years old |
| **Q17** | ChildBody | ‘To what degree did you feel in a child’s body?’ | Not at all/ Very much(-3…3) |
| **Q18** | AdultBody | ‘To what degree did you feel in an adult’s body?’ | Not at all/ Very much(-3…3) |
| **Q19** | Younger | ‘How much younger than your actual age did you feel?’ | Not at all/ Very much(-3…3) |
| **Q20** | Older | How much older than your actual age did you feel?’ | Not at all/ Very much(-3…3) |
| **Q21** | FeltChild | ‘How much did you feel like a child?’ | Not at all/ Very much(-3…3) |

**References**

1. Banakou, D. & Slater, M. Body ownership causes illusory self-attribution of speaking and influences subsequent real speaking. Proc. Natl. Acad. Sci. U. S. A. 111, 17678–17683 (2014).
2. Banakou, D., Groten, R. & Slater, M. Illusory ownership of a virtual child body causes overestimation of object sizes and implicit attitude changes. Proc. Natl. Acad. Sci. 110, 12846–12851 (2013).
3. Banakou, D., Parasuram, D. H. & Slater, M. Virtual Embodiment of White People in a Black Virtual Body Leads to a Sustained Reduction in their Implicit Racial Bias. Front. Hum. Neurosci. 10, 601 (2016).
4. Peck, T. C., Seinfeld, S., Aglioti, S. M. & Slater, M. Putting yourself in the skin of a black avatar reduces implicit racial bias. Conscious. Cogn. 22, 779–787 (2013).

5. Huckvale, M., Leff, J. & Williams, G. Avatar Therapy : : an audio audio-visual dialogue system for treating auditory a hallucinations Department of Speech Department of Mental Health Sciences. in *Interspeech* (2013).

6. Cartei, V. & Reby, D. Effect of formant frequency spacing on perceived gender in pre-pubertal children’s voices. *PLoS One* **8,** 12–18 (2013).

7. Huber, J. E., Stathopoulos, E. T., Curione, G. M., Ash, T. A. & Johnson, K. Formants of children, women, and men: the effects of vocal intensity variation. *J. Acoust. Soc. Am.* **106,** 1532–1542 (1999).

8. Perry, T. L., Ohde, R. N. & Ashmead, D. H. The acoustic bases for gender identification from children’s voices. *J. Acoust. Soc. Am.* **109,** 2988–2998 (2001).

9. Smith, D. R. R. & Patterson, R. D. The interaction of glottal-pulse rate and vocal-tract length in judgements of speaker size, sex, and age. *J. Acoust. Soc. Am.* **118,** 3177–3186 (2005).

10. Valjamae, A., Tajadura-Jimenez, A., Larsson, P., Vastfjall, D. & Kleiner, M. Binaural bone-conducted sound in virtual environments: Evaluation of a portable, multimodal motion simulator prototype. *Acoust. Sci. Technol.* **29,** 149–155 (2008).

11. Pörschmann, C. Influences of bone conduction and air conduction on the sound of one’s own voice. *Acta Acust. united with Acust.* **86,** 1038–1045 (2000).

1. ^^ Ana Tajadura-Jiménez and Domna Banakou contributed equally to this work. [↑](#footnote-ref-2)
2. ^^ [↑](#footnote-ref-3)
